# Cognitive functions and skill level in Brazilian Jiu-Jitsu: an exploratory study using virtual reality

**DOI:** 10.3389/fspor.2025.1738534

**Published:** 2026-01-12

**Authors:** Tomas Peric, Stanislav Novicichin, Łukasz Rydzik, Wojciech Wąsacz, Tadeusz Ambroży, Pavel Ruzbarsky

**Affiliations:** 1 Faculty of Sport, University of Presov, Presov, Slovakia; 2 Faculty of Physical Education and Sport, Charles University, Prague, Czechia; 3 Faculty of Physical Education and Sport, Institute of Sports Sciences, University of Physical Culture, Kraków, Poland

**Keywords:** Brazilian Jiu-Jitsu, combat sports, decision-making, performance level, virtual reality assessment

## Abstract

**Introduction:**

Competitive outcomes in combat sports depend not only on functional preparation but also on athletes’ ability to quickly adapt under pressure and make effective decisions in rapidly changing situations. The use of virtual reality (VR) as a tool for assessing cognitive functions has not yet been thoroughly validated in this context. The VR tasks used in this study do not measure higher-order cognitive functions such as executive function or anticipation; instead, they focus on observable performance metrics, such as reaction time, accuracy, and motor variability. The aim of the present study was to compare the level of cognitive functions in Brazilian Jiu-Jitsu (BJJ) athletes with varying levels of expertise.

**Methods:**

Ten male BJJ practitioners (average age was 27.20 ± 5.63 years) were categorized into professionals (average training experience 11.00 ± 1.41 years; brown/black belts) and semi-professionals (average training experience 5.40 ± 1.14 years, blue/purple belts). VR based cognitive tests assessed reaction time, accuracy, and spatial awareness. The Mann–Whitney *U* test was applied to compare group performance.

**Results:**

The professional group showed higher decision-making accuracy and slower reaction times; however, **these** patterns should be interpreted cautiously, as the study design does not allow attribution to underlying strategies or processes. The semi-professionals, on the other hand, exhibited greater variability in motor responses. Some of the differences between groups reached statistical significance (*p* < 0.05), although these should be interpreted cautiously due to the small sample size. The relationship between experience level and cognitive measures was inconsistent across parameters and should be considered preliminary, as the study was not powered to detect systematic associations.

**Conclusions:**

Professionals appeared to score higher in accuracy-related measures and showed slower reaction times; however, these patterns should be interpreted as preliminary trends rather than evidence of differences in underlying mechanisms or strategic prioritization. Given the very small sample size and the exploratory nature of the study, no conclusions can be drawn about underlying cognitive mechanisms. The findings suggest preliminary, unstable patterns between expertise levels in selected cognitive measures; however, given the small sample size and exploratory design, no causal inferences regarding the influence of experience on cognitive functions can be drawn. The study also suggests that VR may have potential as an assessment tool, though its utility for evaluating BJJ-specific cognitive performance remains unvalidated and should be considered exploratory at this stage.

## 1 Introduction

Brazilian Jiu-Jitsu (BJJ) is a specialized combat sport that integrates physical strength, technical precision, tactical reasoning, and mental resilience. Its structure which includes both stand-up and ground phases and emphasis on submission techniques impose unique physiological and psychological demands, distinguishing it from other martial arts.

Physiologically, BJJ involves a complex and variable load profile. Athletes alternate between intense anaerobic efforts (e.g., during submissions, positional transitions, or escapes) and less intense yet metabolically taxing phases. Lactate levels often exceed 10 mmol/L during matches, indicating a high anaerobic load, while average intensity remains around 60%–70% of VO_2_max (1).

Given this fluctuating nature of internal load, recent studies emphasize the importance of individualized conditioning strategies tailored to the athlete's belt level and match style (gi vs. no-gi), which significantly influence motor performance, fatigue profiles, and recovery demands (2, 3).

A defining feature of BJJ is the sustained isometric effort, especially in the upper body, required to maintain positions or control opponents. A study by Detanico et al. (4) demonstrated that during a simulated Brazilian Jiu-Jitsu tournament, 22 male athletes exhibited significant reductions in countermovement jump height and grip strength, accompanied by progressively increasing ratings of perceived exertion across three successive 7-min matches. Technical and tactical skills are closely linked to athlete development, traditionally represented by belt color. Escobar-Molina et al. (5) noted that as athletes progress (blue to black belt), training shifts from basic techniques to complex transitions and strategic scenarios.

Advanced competitive performance is *often associated* with anticipatory abilities, perception of opponent movement, and rapid adaptation, although the direction and nature of this relationship remain debated. According to Lamas et al. (6), the dynamic structure of no-gi Brazilian Jiu-Jitsu matches highlights how specific transitions especially high-probability, low-risk moves like the back-take can significantly shape athletes' decision-making speed and action selection during competition.

It is important to note that the VR tasks used in this study do not measure executive function, anticipation, or higher-order cognitive control; therefore, the study examines only basic performance metrics such as reaction time, accuracy, and within-subject variability.

Tournament performance in combat sports such as wrestling has been shown to depend not only on physiological readiness but also on athletes' ability to rapidly adapt under pressure and make effective decisions in dynamic scenarios (7). Similarly, research across judo and boxing highlights that higher emotional intelligence and psychological resilience significantly contribute to superior performance during stressful competitive contexts (8).

Mental resilience is as critical as physical capacity. Despite the recognized role of cognitive functions in combat sports, few studies objectively assess their impact in BJJ. The Cognitive-Motor Performance Integration Model (9) suggests that sport performance relies on anticipatory cognition integrated with motor execution under time pressure. However, no studies have applied this or similar frameworks to BJJ. It also remains unclear whether virtual reality (VR) can effectively assess or develop these skills. The extent to which cognitive abilities differ between professional and semi-professional BJJ athletes and how these differences relate to performance remains unexplored.

Recent research in Brazilian Jiu-Jitsu (BJJ) highlights increasing interest in using virtual reality (VR) for personalized training and psychological development. Blick (10) reports that empathy, mindfulness, and resilience improve with belt level, suggesting VR could help cultivate these traits. Power (11) shows that BJJ enhances mental health in law enforcement, indicating VR may offer a safe tool for stress adaptation. Dos Santos et al. (12) link belt rank to performance differences in elite female athletes, supporting the creation of skill-specific VR training models.

Although constructs such as anticipation, situational awareness, and cognitive control are frequently discussed in expertise literature, the present study did not measure these abilities and cannot make any claims regarding them.

The aim of the present study was to compare the level of selected cognitive functions (reaction time, decision-making accuracy, and motor response variability) in Brazilian Jiu-Jitsu athletes with different levels of expertise (professionals and semi-professionals). Due to the small sample size (*n* = 10), the focus is not on generalizability, but on identifying preliminary trends that may guide future research. Drawing on the CMPI framework, performance is viewed as the interplay of reaction time, decision-making under pressure, and motor variability.

The specific objectives are:

Objectively assess cognitive functions (reaction time, decision-making, motor variability) in professional vs. semi-professional BJJ athletes.
Identification of quantitative and qualitative differences in cognitive profiles between groups with different levels of athletic expertise.
Preliminary observation of the co-occurrence between training and sports experience level and cognitive functioning, using a custom virtual reality (VR)-based protocol.
Evaluation of the applicability of VR as a tool for assessing and potentially training cognitive functions in the context of combat sports, specifically BJJ.

Research question and hypothesis:

Assuming that athletic performance in BJJ results from the interaction of cognitive and motor domains as outlined in models like CMPI this study asks: To what extent do selected cognitive functions (reaction time, decision-making accuracy, motor variability), assessed using virtual reality (VR), differ between Brazilian Jiu-Jitsu (BJJ) athletes at different levels of expertise (professional and semi-professional)?

Based on this, we propose the following hypotheses:

H1: Professional BJJ athletes will show faster reaction times and lower motor variability than semi-professionals.
H2: Professional athletes will exhibit higher decision accuracy than semi-professional athletes when performing time-pressured VR decision tasks.
H3: The VR-based environment will enable exploratory assessment of cognitive differences between groups and may indicate potential avenues for future application in combat sports training.

## 2 Methods

### 2.1 Ethical approval

The studies involving humans were approved by Ethics Committee of the Faculty of Physical Education and Sport, Charles University (UK FTVS), under reference number 248/2023. The studies were conducted in accordance with the local legislation and institutional requirements. The studies were conducted in accordance with the Helsinki Declaration requirements. The participants provided their written informed consent to participate in this study.

### 2.2 Study design

This exploratory cross-sectional study was conducted in the VR laboratory at SENSE Arena, Faculty of Physical Education and Sport, Charles University. The aim was to assess cognitive functions in professional and semi-professional BJJ athletes under standardized conditions. Given the small sample (*n* = 10), findings are indicative rather than generalizable.

### 2.3 Sample size calculation

The determination of the sample size was guided by an *a priori* power analysis performed using GPower 3.1 software. Assuming a medium effect size (Cohen's *d* = 0.5) for differences in cognitive performance between professional and semi-professional athletes, a two-tailed Mann–Whitney *U* test with an alpha level of 0.05 and statistical power of 0.80 would require a minimum of 21 participants per group. An *a priori* power analysis indicated that at least 21 participants per group would be required to detect medium effects with adequate statistical power (*α* = .05, 1 − *β* = .80). Because recruiting professional BJJ athletes matching the inclusion criteria was highly constrained, the study proceeded with a convenience sample of 5 athletes per group. As a result, the study is severely underpowered, and any statistically significant findings must be viewed as unstable and potentially sample-specific.

### 2.4 Participants characteristics

Ten male athletes aged 20–35 (*x˜* = 26.20 ± 5.03) and with average training experience 8.20 ± 3.19 were deliberately divided into two groups by quantifiable competitive criteria. Professionals (*n* = 5; average age was 27.20 ± 5.63 years): ≥10 years of BJJ experience (*x˜* = 11.00 ± 1.41), brown (*n* = 3)/black (*n* = 2) belt, recent international medal (IBJJF). Semi-professionals (*n* = 5; 25.20 ± 4.76 years): 4–7 years of experience (x˜ = 5.40 ± 1.14), blue (*n* = 3)/purple (*n* = 2) belt, regional/national-level activity. All were right-handed, with comparable body types and autonomous motor skill execution. Informed consent was obtained. Information on chronological age, training experience, activity level, and competitive history was gathered using a diagnostic survey method, implemented through direct interviews with the athletes and their coaching staff. The study was conducted during the preparatory phase of the training cycle. The inclusion criteria for the study were as follows: no history of serious injuries, a minimum of four years of training experience, right-handedness and active participation in competitions. The exclusion criteria included violation of any of the above conditions, current injuries or medical conditions that could affect participation in the study, lack of informed consent, absence of competitive experience, previous VR training experience and the use of performance-enhancing substances.

### 2.5 Testing procedure

All participants were tested individually in a controlled laboratory environment between 14:00 and 16:00 to minimize diurnal variation. Task order was fixed across participants due to software constraints, and all athletes were instructed to refrain from strenuous training for 24 h prior to testing to minimize fatigue-related variability. No participant reported prior experience with VR training.

Participants were familiarized with the VR interface before completing a battery of standardized cognitive tests via SenseArena software (SenseArena s.r.o., Prague, Czech Republic) using the HTC Vive Pro 2 headset. Although the SenseArena tests were not designed specifically for BJJ, they were selected because they target general cognitive components—such as stimulus discrimination, divided attention, and rapid decision-making—that are theoretically relevant to open-skill combat scenarios. Importantly, no peer-reviewed data are available regarding the reliability, validity, or psychometric properties of these tasks in combat sports or BJJ populations, and the platform does not provide grappling-specific stimuli (e.g., positional cues, opponent pressure, movement patterns). As a result, the ecological validity and measurement precision of these tests for BJJ remain limited. Therefore, the present results should be interpreted strictly as indicators of general cognitive tendencies rather than sport-specific cognitive performance, and any extrapolation to real-world BJJ contexts should be considered preliminary.

### 2.6 Cognitive variables

All participants were tested individually in a controlled laboratory environment between 14:00 and 16:00 to minimize diurnal variation. Task order was fixed across participants due to software constraints, and all athletes were instructed to refrain from strenuous training for 24 h prior to testing to minimize fatigue-related variability. No participant reported prior experience with VR training.

Participants were familiarized with the VR interface before completing a battery of standardized cognitive tests via SenseArena software (SenseArena s.r.o., Prague, Czech Republic) using the HTC Vive Pro 2 headset. Although the SenseArena tests were not designed specifically for BJJ, they were selected because they target general cognitive components—such as stimulus discrimination, divided attention, and rapid decision-making—that are theoretically relevant to open-skill combat scenarios. Importantly, no peer-reviewed data are available regarding the reliability, validity, or psychometric properties of these tasks in combat sports or BJJ populations, and the platform does not provide grappling-specific stimuli (e.g., positional cues, opponent pressure, movement patterns). As a result, the ecological validity and measurement precision of these tests for BJJ remain limited. Therefore, the present results should be interpreted strictly as indicators of general cognitive tendencies rather than sport-specific cognitive performance, and any extrapolation to real-world BJJ contexts should be considered preliminary.

All tests originated from the SenseArena platform, which has internal validation but lacks peer-reviewed BJJ-specific reliability data. This remains a key limitation.

### 2.7 Equipment and study flowchart

Testing employed HTC Vive Pro 2 headsets (HTC Corporation, Taoyuan City, Tajwan) and SenseArena software (Sense Arena s.r.o., Prague, Czech Republic). Although the VR tasks include general perceptual-motor elements common to combat sports, they do not simulate grappling-specific interactions such as positional transitions, grip sequences, or opponent pressure. This lack of contextual specificity reduces the ecological validity of the assessment and limits the degree to which performance in these VR tasks can be extrapolated to real BJJ decision-making or tactical behavior. Future protocols should integrate sport-specific stimuli to improve ecological validity.

**Figure 1:**
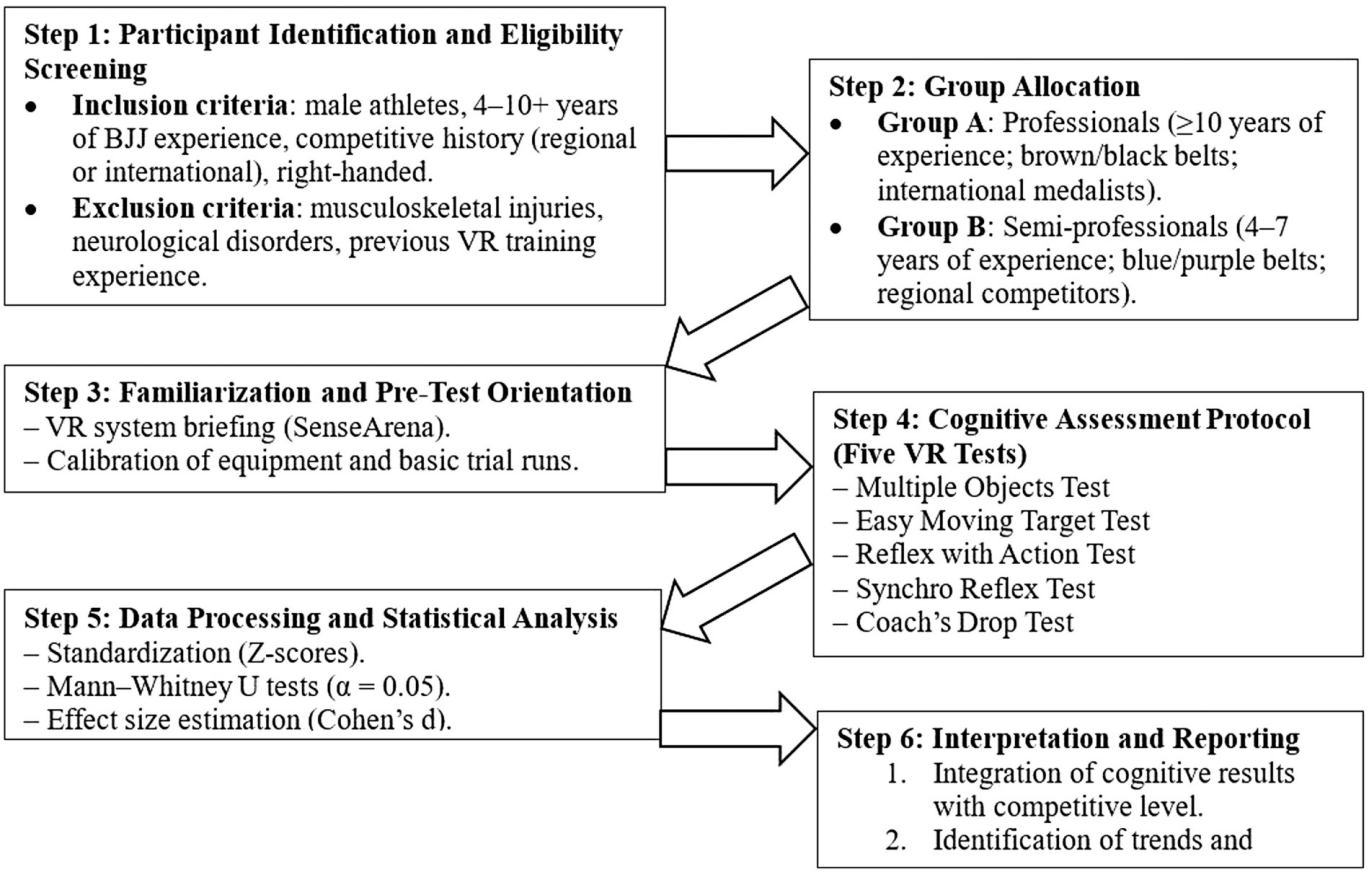
Study procedure diagram.

The overall study procedure is summarized in Figure 1. This flow diagram provides a structured overview of all methodological steps, from participant screening and group allocation through virtual reality familiarization, cognitive testing, and statistical analysis. Presenting these stages in a visual format enhances clarity and allows readers to follow the study design more intuitively.

### 2.8 Statistical analysis

Descriptive statistics were calculated for all variables. Group comparisons were conducted using non-parametric Mann–Whitney *U* tests, as the small sample size (*n* = 5 per group) precluded assumptions of normality. For each comparison, we report the *U* statistic, standardized *Z* value, exact *p*-value, and corresponding effect size (*r*), in line with recommended practice for small-sample non-parametric testing. 

To enable exploratory comparisons across heterogeneous VR task metrics, raw values were also standardized using *Z*-scores computed across the entire sample (*N* = 10). These standardized values serve purely descriptive purposes and should be interpreted with caution, as *Z*-scores are highly sensitive to outliers and may exaggerate apparent between-group differences.

Cohen's *d* was additionally computed for descriptive effect size estimation, but given the small sample and non-parametric distributions, *r*-values from the Mann–Whitney tests are considered more informative. Standard deviations (SDs) were used to characterize within-group variability.

Because of the small sample size, raw medians and interquartile ranges (IQRs) for all cognitive variables are included in Supplementary Table S1 to allow transparent evaluation of group distributions. All analyses were conducted using SPSS (IBM SPSS Statistics, Version 29.0.2.0) and Microsoft Excel (Version 2511). The statistical significance level was set at *p* < 0.05, but given the exploratory nature of the study, *p*-values should not be interpreted as confirmatory evidence.

Given the exploratory nature of this study, no correction for multiple comparisons was applied. As a result, the probability of Type I error is increased, and statistically significant findings should be interpreted with caution, particularly in the context of the very small sample size.

## 3 Results

Cognitive performance was assessed across five virtual reality-based tasks, each targeting specific components such as reaction time, decision-making accuracy, attention, and motor coordination (see Table 1). Then the results were presented by test type, comparing professional and semi-professional BJJ athletes. To improve transparency, all raw medians and interquartile ranges (IQRs) for the 14 partial variables are reported in Supplementary Table S1. Because the study included multiple statistical comparisons without correction procedures, and given the small sample size (*n* = 5 per group), any statistically significant *p*-values may be unstable and should not be interpreted as confirmatory evidence. While only one test yielded statistically significant group differences, several consistent trends were observed, particularly in favor of professionals in cognitively demanding domains (see Table 2). Due to the small sample and exploratory design, findings should be interpreted cautiously.

**Table 1:** Virtual reality-based cognitive tests and their key performance indicators.

Test	Key variables	Purpose
Multiple objects	Reaction time, decision time	Multitasking and threat detection
Easy moving target	Caught balls, release time	Responsiveness to dynamic stimuli
Reflex with action	Reaction/motor time, accuracy	Simulated defensive-reactive tasks
Synchro reflex	Reaction/motor time, task success	Sensorimotor coordination
Coach's drop	Efficiency, concentration	Guided decision-making accuracy

**Table 2:** Comprehensive results table: *U*, *Z*, exact *p*, and effect sizes.

Groups/Outcome	*U*	*Z* (no CC)	*p*_exact 1-sided	*p*_exact 2-sided	*p*_asymp 2-sided	Cohen's *r*	Cohen's *d*
1—Reaction time (ms)	3	−1.974	0.0278	0.0556	0.0472	−0.624	0.92
1—AVG time to correct decision (ms)	0	−2.613	0.0079	0.0159	0.0251	−0.826	3.96
2—Caught balls (%)	7	−1.047	0.0952	0.1905	0.0432	−0.331	0.28
2—Release time (ms)	11	−0.104	0.376	0.754	0.285	−0.033	0.04
3—Covered game field (%)	11	−0.104	0.376	0.754	0.689	−0.033	0.21
3—Median motor time (ms)	11	−0.104	0.376	0.754	0.371	−0.033	0.39
3—Median reaction time	5	−1.567	0.0476	0.0952	0.169	−0.496	1.03
3—Correct attempts (%)	11	−0.104	0.376	0.754	0.332	−0.033	0.87
4—Median reaction time	9	−0.627	0.0952	0.1905	0.347	−0.199	0.66
4—Successful tasks (%)	10	−0.418	0.310	0.619	0.554	−0.132	0.30
4—Median motor time	5	−1.567	0.0476	0.0952	0.093	−0.496	0.18
5—Efficiency (%)	4	−1.774	0.0278	0.0556	0.236	−0.561	I.22
5—Concentration (%)	4	−1.774	0.0278	0.0556	0.036	−0.561	I.35
5—Reaction time (ms)	9	−0.627	0.0952	0.1905	0.288	−0.199	−0.60

For Groups/Outcome The number in the first column indicates the test group for each outcome: 1, Multiple Objects Test; 2, Easy Moving Target Test; 3, Reflex with Action Test; 4, Synchro Reflex Test; 5, Coach's Drop Test. Each outcome is listed with its group number to show the test battery from which it originates. For the Statistical Summary Table. *U***—**Mann–Whitney *U* statistic representing the summed ranks of the lower-ranked group. *Z* (no CC)—standardized *Z*-value computed from the *U* **statistic** without continuity correction, as recommended when exact *p*-values are available. *p*_exact 1-sided—exact one-tailed *p*-value based on the full permutation distribution of U. *p*_exact 2-sided—exact two-tailed *p*-value for the Mann–Whitney test, reported as the primary significance estimate for small samples. *p*_asymp 2-sided—asymptotic two-tailed *p*-value from the normal approximation, shown for comparison with standard software output. Cohen's *r*—rank-based effect size computed as *Z*/√*N*, where *N* is the total number of observations. Cohen's *d*—standardized mean difference based on group means and pooled standard deviation derived from the reconstructed datasets.

### 3.1 Multiple objects test

This test evaluated Reaction Time and Average Time to Correct Decision.

**Reaction Time (RT):** Professionals had a longer mean MT (*M* = 2,801.2 ms; SD = 1,690.24) than semi-professionals (*M* = 1,691.48 ms; SD = 253.96), with a statistically significant difference (*p* = 0.047; *d* = 0.92). Given the extremely small sample size (*n* = 5 per group), these observed differences—including large effect sizes—must be interpreted with caution, as they may reflect sample-specific variability rather than systematic expertise-related effects. In addition, this comparison is statistically underpowered and no correction for multiple comparisons was applied, increasing the likelihood of a Type I error. Importantly, no inference can be made regarding underlying cognitive strategies (e.g., delayed responding or “strategic processing”), as the study did not include any measures capable of assessing such mechanisms. Alternative explanations such as individual variability or age differences remain equally plausible.
**Average Time to Correct Decision:** Professionals achieved significantly faster decision times (*M* = 68.6 ms; SD = 15.63) than semi-professionals (*M* = 228.8 ms; SD = 54.86), with a very large effect (*p* = 0.025; *d* = –3.96). Although professionals showed faster decision-making times, this finding must be interpreted with caution. In addition, this comparison is statistically underpowered and no correction for multiple comparisons was applied, increasing the likelihood of a Type I error. The extremely small sample size, high variability, and absence of sport-specific task validity may inflate the observed effect size. Importantly, the study did not include any measures designed to assess cognitive efficiency, processing speed, or executive control mechanisms; therefore, no inferences about these constructs can be drawn from the present data. Overall the observed difference should be considered a preliminary pattern only. The VR tasks assess decision accuracy and reaction time but do not provide information about decision-making quality, strategy, or efficiency; therefore, interpretations must be limited to the observed performance metrics.

**Summary:** Professionals showed shorter decision times but longer reaction times; however, these patterns are preliminary and may reflect sampling variability. Due to the small sample size and uncontrolled confounders (e.g., age, experience), results should be viewed as preliminary.

### 3.2 Easy moving target test

This test included Caught Balls (%) and Release Time (ms).

**Caught Balls (%):** Professionals showed a higher success rate (*M* = 49.2%; SD = 6.42) than semi-professionals (*M* = 38.4%; SD = 54.86), with statistical significance (*p* = 0.0432) but only a small effect size (*d* = 0.28). However, the extreme variability in the semi-professional group, combined with the very small sample size (*n* = 5 per group), indicates substantial measurement instability. In addition, this comparison is statistically underpowered and no correction for multiple comparisons was applied, increasing the risk of a Type I error. As such, the apparent group difference may be partly or entirely attributable to noise, and the effect size is likely inflated. Given also that this VR task has limited ecological validity for BJJ, the practical meaningfulness of this result remains highly uncertain.
**Release Time (ms):** Professionals had a slightly longer mean release time (*M* = 767.2 ms; SD = 259,8) than semi-professionals (*M* = 749.4 ms; SD = 269,6), but the difference was non-significant (*p* = 0.285; *d* = 0.04). Given the very small sample size (*n* = 5 per group), non-significant results cannot be interpreted as evidence of no group difference, as the study is substantially underpowered to detect small or moderate effects. Therefore, this outcome should be interpreted as inconclusive rather than indicative of task insensitivity or a lack of BJJ-related influence.

**Summary:** While professionals obtained higher catch rates, interpretation is limited by variability, small sample size, and task specificity. Findings should be considered tentative pending replication with sport-specific stimuli and better-controlled conditions.

### 3.3 Reflex with action test

Four variables were analyzed: Covered Game Field (%), Median Motor Time (ms), Median Reaction Time (ms), and Correct Attempts (%). Although several effect sizes appeared moderate to large, their interpretability is limited by the high inter-individual variability and the extremely small sample size, both of which can artificially inflate standardized effect estimates. However, all interpretations must be considered preliminary given the limited sample size and potential influence of uncontrolled variables such as fatigue, prior exposure to VR, or inter-individual learning rates

**Covered Game Field (%):** Professionals covered a marginally larger field area (*M* = 29.8%) than semi-professionals (*M* = 28.69%), with a small effect (Cohen's *d* = 0.21). This minimal difference is unlikely to reflect a systematic group effect and may instead be attributable to inter-individual variability or measurement noise. Because the VR task does not assess constructs such as spatial awareness, no psychological interpretation can be drawn from this outcome.
**Median Motor Time (median MT) (ms):** Professionals showed a shorter motor execution time (*M* = 960.41 ms) than semi-professionals (*M* = 1,047.49 ms), with a small-to-moderate effect size (Cohen's *d* = −0.39). Although professionals displayed shorter motor times, this cannot be interpreted as evidence of superior motor efficiency, as the study did not include any measures designed to assess motor efficiency or execution quality. Given the very small sample size and substantial inter-individual variability, this difference should be considered inconclusive rather than indicative of a systematic group effect.
**Median Reaction Time (ms):** A relatively large numerical difference was observed in cognitive reaction time, with professionals responding more rapidly (*M* = 232.2 ms) than semi-professionals (*M* = 293.0 ms), corresponding to a large effect size (Cohen's *d* = –1.03). Although this difference was not statistically significant (*p* = 0.169), the result should be interpreted as an exploratory observation only. Given the very small sample size, the lack of BJJ-specific validation of the VR tasks, and uncontrolled confounders such as age, fatigue, or task familiarity, the study is underpowered to determine whether this numerical advantage reflects a true group effect. The non-significant outcome therefore cannot be taken as evidence for or against expertise-related differences in reaction time.
**Correct Attempts (%):** Professionals attained a slightly higher accuracy rate (*M* = 98%) than semi-professionals (*M* = 95.6%), with a large descriptive effect size (Cohen's *d* = 0.87). However, because this difference was not statistically significant and the sample size is extremely small (*n* = 5 per group), it cannot be interpreted as evidence of superior performance consistency. The observed numerical gap may reflect individual variability or familiarity with the task rather than a systematic group effect. Since the VR task measures accuracy but not execution stability or attentional control, no psychological interpretation can be drawn from this result.

**Summary:** Overall, results from the Reflex with Action Test indicate only numerical differences in the professional group that require cautious interpretation, as these patterns may reflect sampling variability rather than systematic expertise-related effects. The absence of statistically significant differences, combined with the study's methodological constraints particularly the small and homogeneous sample and unvalidated BJJ specificity of the test limits the strength of any conclusions regarding performance-level differences. Future research should incorporate larger samples, objective confounder controls, and longitudinal tracking to validate these preliminary observations.

### 3.4 Synchro reflex test

Three variables were examined: Median Reaction Time (ms), Successful Tasks (%), and Median Motor Time (ms). None of the group differences reached statistical significance (*p* > 0.05), though effect sizes suggested minor trends.

**Median Reaction Time (ms):** Semi-professional fighters demonstrated slightly faster median RT (*M* = 396.0 ms) compared to professionals (*M* = 418.0 ms), although this difference occurred alongside notably higher variability in the semi-professional group (SD = 39.3 vs. 26.0). The effect size was moderate (Cohen's *d* = 0.66), but given the very small sample size (*n* = 5 per group), this value should be regarded as descriptive rather than indicative of a stable performance trend. The Mann–Whitney test (*p* = 0.347) did not yield statistical significance, and with such limited statistical power, non-significant findings cannot be interpreted as evidence for or against group differences. Therefore, this numerical pattern should be considered inconclusive rather than reflective of genuine differences in reaction capabilities.
**Successful Tasks (%):** Professionals achieved a marginally higher success rate (*M* = 92.2%) than semi-professionals (*M* = 90.4%), with slightly lower variability (SD = 5.68 vs. 6.42). The effect size was small (Cohen's *d* = 0.30), and given the very small sample size, this value should be interpreted as a descriptive pattern only. The Mann–Whitney test (*p* = 0.554) showed no statistically significant difference, and with such limited statistical power, non-significant findings cannot be taken as evidence for or against group differences. Therefore, this numerical difference should be considered inconclusive, and no interpretation regarding performance stability or underlying psychological factors can be drawn from this outcome.
**Median Motor Time (ms):** Semi-professionals exhibited shorter median motor execution times (*M* = 502.6 ms) compared to professionals (*M* = 596.0 ms), but with substantially higher variability (SD = 749.31 vs. 145.16). The extremely large SD in the semi-professional group relative to the mean indicates that task performance was highly unstable and likely dominated by measurement noise. As such, the difference in group means cannot be interpreted as a meaningful performance distinction. The corresponding effect size was small (Cohen's *d* = 0.18), reflecting a negligible descriptive difference. The Mann–Whitney test (*p* = 0.093) did not reach statistical significance, and with the very small sample size (*n* = 5 per group), non-significant findings cannot be taken as evidence for or against group differences. Therefore, this numerical pattern should be considered inconclusive, and no interpretation regarding motor execution quality or underlying mechanisms can be drawn from this outcome.

**Summary:** While no statistically significant differences emerged, professionals showed numerically higher accuracy, whereas semi-professionals showed slightly faster but more variable responses. Given the extremely small sample size (*n* = 5 per group) and high variability across participants, these results should be considered purely preliminary. Observed differences—including moderate effect sizes—may be inflated by sampling noise and require confirmation in substantially larger and better-controlled cohorts.

### 3.5 Coach's drop test

This test measured Efficiency, Concentration, and Reaction Time. While only concentration differences were statistically significant, effect sizes suggested practical trends. Due to the small sample and lack of controls (e.g., cognitive baseline, psychological state), findings remain tentative.

**Efficiency (%):** Although professionals obtained higher efficiency scores (91.20% vs. 79.20%), this difference was not statistically significant (*p* = 0.236), and the very large effect size (Cohen's d = 1.22) is likely inflated due to high within-group variability and the extremely small sample size (*n* = 5 per group). As such, this numerical difference should be interpreted as a descriptive pattern rather than as evidence of superior task execution or performance efficiency. Moreover, the VR task does not assess constructs such as execution quality or cognitive efficiency, and non-significant findings in an underpowered design cannot be taken as evidence for or against group differences.
**Concentration (%):** Although professionals scored higher on the concentration metric (66.00% vs. 18.2%; *p* = 0.036; d = 1.35), this difference must be interpreted with extreme caution. The very small sample size (*n* = 5 per group), high inter-individual variability, and limited ecological validity of the VR task substantially increase the likelihood that this effect size is inflated. In addition, this comparison is statistically underpowered and no correction for multiple comparisons was applied, increasing the risk of a Type I error. Therefore, this result should be interpreted as a preliminary and potentially sample-specific observation rather than as evidence of superior attentional performance or underlying psychological mechanisms. Given these constraints, no inference can be made about factors such as focus, motivation, or task familiarity, which were not measured in this study.
**Reaction Time (ms):** Group means were similar between professionals (315.2 ms) and semi-professionals (322.8 ms), and the difference was not statistically significant (*p* = 0.288). Although the effect size (Cohen's d = –0.60) suggests a numerical difference in variability, this value should be interpreted as descriptive only, as the extremely small sample size (*n* = 5 per group) renders the result underpowered and highly sensitive to individual fluctuations. Non-significant findings in this context cannot be taken as evidence for or against group differences, and the VR task does not assess constructs such as performance consistency or stability. Therefore, no inference can be made regarding experience-related differences or underlying mechanisms in reaction time performance.

**Summary:** Professionals showed higher scores on the concentration metric and efficiency score; however, these values do not reflect psychological constructs such as focus or cognitive efficiency and must be interpreted cautiously.

Following the presentation of all outcome-specific results, the complete set of statistical outputs for every test battery included in this study is summarized below. Table 2 reports the *U* statistic, *Z*-value (calculated without continuity correction), exact *p*-values, asymptotic *p*-values, and both effect size indices (Cohen's r and Cohen's d) for all measured outcomes.

An overview of all outcomes and their corresponding test groups is presented in Table 2.

### 3.6 Integrated analysis across tests

Table 3 presents an exploratory comparison of all five test batteries using standardized *Z*-scores. Given the small sample size (*N* = 10) and variability in several tasks, these *Z*-scores should be interpreted cautiously, as their magnitude may reflect scaling artefacts rather than stable performance differences. A statistically significant difference emerged only in the **Multiple Objects Test** (*p* = 0.036), where professionals obtained higher scores in this sample. Although professionals obtained higher composite *Z*-scores, this pattern cannot be interpreted as evidence of superior multitasking or decision-making. *Z*-scores calculated on such a small sample (*N* = 10) are highly sensitive to outliers and variability, and may inflate apparent between-group differences. With such a small sample (*n* = 10), *Z*-scores are highly sensitive to outliers and measurement variability, and the differences may largely reflect scaling artefacts rather than true performance distinctions. The non-sport-specific nature of the VR tasks further limits the interpretability of these standardized comparisons.

**Table 3:** Integrated analysis of all five test batteries using mean *Z*-scores.

Test	*Z*-scores (Professionals)	*Z*-scores (Semi-professionals)	Mann–Whitney Test (*p*-value)
Multiple objects	0.128	0.064	0.036*
Easy moving target	0.246	0.325	0.086
Reflex with Action	0.188	0.213	0.249
Synchro reflex	0.890	0.812	0.284
Coach's drop	0.682	0.579	0.183

* Level of significance statistically significant values (*p* < 0.05).

Although other tests did not reach statistical significance, several trends are notable. **Easy Moving Target:** semi-professionals scored slightly higher (*Z* = 0.325 vs. 0.246), possibly due to faster—but less controlled—responses. However, high variability limits interpretability. **Reflex with Action:** both groups achieved similar results, suggesting comparable performance in integrated sensorimotor tasks. Minor differences may reflect noise rather than systematic effects. **Synchro Reflex:** high and closely matched scores (professionals *Z* = 0.89; semi-professionals *Z* = 0.812) suggest this test may reflect individual motor coordination predispositions rather than training level. And the **Coach's Drop:** professionals scored higher (*Z* = 0.682 vs. 0.579), though without statistical significance. This mirrors the numerical pattern observed earlier in the concentration variable, but cannot be interpreted as evidence of attentional differences.

**Summary:** While only one test showed statistical significance, professionals generally obtained numerically higher scores in several cognitive metrics, particularly in attention and decision-making. Nevertheless, due to small sample size, homogeneity, and the non-BJJ-specific nature of the tasks, these findings should be interpreted as exploratory. Future research with larger, stratified samples and sport-specific stimuli is needed to validate these preliminary trends.

## 4 Discussion

This study found moderate to large effect sizes in cognitive domains such as decision-making and concentration, with statistically significant differences only in the Multiple Objects Test. It is important to emphasize that the present study cannot determine whether any observed differences reflect genuine expertise-related cognitive advantages. The results represent preliminary associations only and should not be interpreted as evidence of underlying cognitive mechanisms or strategies.

Although professionals tended to score higher in some cognitively demanding tasks, these observations should be treated as preliminary patterns rather than robust expertise-related effects. It should also be noted that the VR tasks used in this study assess decision accuracy and reaction time but do not provide information about decision-making quality, strategy, or efficiency. Accordingly, interpretations must remain limited to the observed performance metrics. The small sample, high inter-individual variability, age and experience differences, and limited ecological validity of the VR tests substantially constrain any strong interpretation.

Because the VR tasks do not measure psychological constructs such as focus, anticipation, or cognitive control, any interpretation invoking these mechanisms would be speculative and is therefore avoided.

Furthermore, the VR tasks used in this study are not designed to assess higher-order cognitive constructs such as advanced cognitive processing, executive functioning, or strategic decision-making. As such, the present findings should not be interpreted in terms of underlying cognitive mechanisms.

Although these findings cannot be interpreted in terms of anticipatory processing or attentional mechanisms, prior research in expertise suggests that such constructs may play a broader role in combat sports performance. For example, Lucia et al. (9) demonstrated improved cognitive efficiency after integrated cognitive-motor training in athletes. Similarly, Voss et al. (13) showed that elite athletes outperform novices in executive function and cognitive control under pressure.

However, our results must be interpreted with caution. The small, homogeneous sample, lack of sport-specific VR tasks, and uncontrolled confounding factors (e.g., age, experience, fatigue) limit generalizability. The observed performance gap may not reflect expertise alone and could instead be influenced by uncontrolled factors such as age, fatigue, motivation, or task familiarity (7).

Future research should employ larger samples, longitudinal designs, and ecologically valid VR simulations tailored to BJJ, as suggested by Neumann et al. (14). Establishing standardized athlete classifications and validating test batteries in combat sport contexts is also essential.

These findings have several important implications for both applied practice and future research. If any cognitive differences exist between the groups, they appear only as tentative trends that require verification. Due to methodological limitations, the present findings cannot demonstrate that cognitive factors differentiate performance levels in BJJ. This aligns with contemporary neurocognitive models of expertise, which emphasize anticipatory processing, rapid situation assessment, and adaptive motor responses as key determinants of competitive success in open-skill sports.

Given the exploratory design and methodological constraints, VR-based cognitive profiling cannot currently be recommended for talent identification or training prescription. Its potential utility requires validation with larger, sport-specific datasets.

From an applied perspective, VR-based cognitive assessment may eventually contribute to athlete development programs, but its applied use remains speculative until validated with sport-specific tasks and larger samples. For instance, athletes demonstrating slower decision times or greater motor variability could benefit from specific drills designed to enhance selective attention, multitasking, and stress inoculation. Additionally, VR environments allow safe replication of high-pressure competitive scenarios, potentially accelerating the acquisition of automatized responses without the physical strain of repeated sparring sessions.

Another potential implication concerns future research on athlete development rather than talent identification. Although exploratory numerical differences were observed in some cognitive–motor metrics, the present study does not provide evidence that VR-based assessments can detect traits associated with elite performance. Such applications would require validated sport-specific tasks, longitudinal data, and substantially larger samples. In the context of BJJ—where athlete evaluation often relies on belt progression and competition results—future research may nevertheless benefit from examining how cognitive–motor characteristics interact with technical and tactical development.

Nonetheless, the current study's exploratory nature underscores the need for methodological refinement. The lack of BJJ-specific stimuli in VR tasks likely underestimated ecological validity; incorporating realistic combat scenarios such as guard passes, sweeps, and submission chains could provide more accurate insights into the interplay between cognition and technical execution. Moreover, longitudinal designs tracking cognitive development across belt levels would clarify whether observed differences are a cause or consequence of expertise.

Finally, the psychological dimension warrants deeper exploration. Constructs such as resilience, emotional regulation, and situational awareness factors shown to influence combat sport outcomes may interact synergistically with cognitive processing, offering a more comprehensive understanding of performance optimization. Integrating psychophysiological metrics (e.g., heart rate variability, EEG markers of anticipation) into VR protocols could further enrich athlete assessment and training paradigms.

### 4.1 Limitations of the study

This study is limited by its small sample size, which constrained statistical power relative to an *a priori* calculation in G*Power 3.1 indicating 21 participants per group. The use of only five participants per group underscores the exploratory nature of the findings and highlights the need for replication in larger samples. Furthermore, the scarcity of professional male BJJ athletes in the Czech Republic restricted recruitment and limits generalizability. Furthermore, the validity of the VR tests for BJJ remains unestablished. While the tasks capture generic cognitive domains, their lack of sport-specific cues means that high task performance may not correspond to grappling decision-making in realistic contexts. Therefore, the present findings should not be used to infer sport-specific cognitive advantages without further validation.

Because *Z*-scores were calculated across all participants in a very small sample, their stability is limited and they may exaggerate relative differences between groups. Therefore, the standardized scores presented here should be interpreted purely as exploratory visualizations.

## 5 Conclusions

This exploratory study aimed to assess and compare the level of selected cognitive functions in Brazilian Jiu-Jitsu (BJJ) athletes with different levels of expertise, using cognitive tests conducted in a virtual reality (VR) environment.

H1 was partially supported: professionals tended to show numerically lower variability in some measures, no consistent or statistically significant advantage was found in reaction time or motor variability. H2 received partial support—a significantly higher decision-making accuracy was observed in the professional group, which may reflect a numerical difference in accuracy under time pressure rather than systematic processing differences. H3 received partial support: the VR environment enabled exploratory measurement of basic cognitive-motor metrics, but its capacity to differentiate levels of cognitive functioning remains unvalidated and should be interpreted cautiously.

Overall, the findings should be interpreted as preliminary and hypothesis-generating. Due to the small sample size, high measurement variability, and limited ecological validity of the VR tasks, no conclusions can be drawn regarding whether attentional or decision-making factors truly differentiate performance levels in BJJ. Any apparent group patterns should be considered unstable and potentially sample-specific rather than indicative of systematic expertise-related differences. However, given the small and homogeneous sample, these findings should be regarded as preliminary and hypothesis-generating rather than confirmatory. Future studies with larger, stratified cohorts and validated, domain-specific cognitive tools are necessary to verify and extend these initial observations.

### 5.1 Practical implications

The results of this study provide preliminary insights that may inform future research and long-term methodological development in BJJ and other combat sports. However, given the exploratory design, small sample size, and limited ecological validity of the VR tasks, the present findings should not be used to guide athlete selection, talent identification, or individualized training prescriptions.

First, although professionals obtained numerically higher scores in some VR-based cognitive metrics, these differences were inconsistent across tasks and cannot be interpreted as cognitive advantages or performance determinants. At best, they highlight domains—such as reaction time and decision accuracy—that may warrant further investigation in more robust, sport-specific studies.

Second, while VR technology offers a controlled and low-risk environment for cognitive-motor assessment, the tasks used in this study do not simulate combat pressure or measure psychological constructs such as resilience, anticipation, or adaptive decision-making. Therefore, any application of VR to training design remains speculative and requires validation with larger samples and sport-specific task paradigms.

Third, although the exploratory numerical patterns observed in this study may encourage further investigation into how cognitive–motor performance relates to athlete development, the present findings do not provide evidence for talent identification, detection of stable cognitive characteristics, or prediction of competitive outcomes. Any such applications would require longitudinal research, validated sport-specific cognitive measures, and substantially larger samples to establish reliability and practical relevance.

Finally, the integration of cognitive and motor assessments represents a promising direction for interdisciplinary research. Future work combining VR-based performance metrics with validated psychophysiological measures may help clarify how cognitive processes interact with technical and tactical skill execution, but these approaches remain beyond the scope of the present study.

## Data Availability

The original contributions presented in the study are included in the article/Supplementary Material, further inquiries can be directed to the corresponding author.

## References

[1] Andreato LV Franchini E de Moraes SM Pastório JJ da Silva DF Esteves JV Physiological and technical-tactical analysis in Brazilian Jiu-Jitsu competition. Asian J Sports Med. (2013) 4(2):137–43. 10.5812/asjsm.34496 23802056 PMC3690734

[2] Coswig VS Bartel C Del Vecchio FB. Brazilian Jiu-Jitsu matches induced similar physiological and technical-tactical responses in gi and nogi conditions. Archives of Budo. (2018) 14:291–301.

[3] Wąsacz W. Brazilian Jiu-Jitsu gi vs nogi—a comparative analysis of the motor fitness level of athletes specializing in different fighting formats. J Kinesiol Exerc Sci. (2025) 36(110):1–10. 10.5604/01.3001.0055.0944

[4] Detanico D Dellagrana RA Athayde MSDS Kons RL Góes A. Effect of a Brazilian Jiu-Jitsu-simulated tournament on strength parameters and perceptual responses. Sports Biomech. (2016) 16(1):115–26. 10.1080/14763141.2016.1206143 27435030

[5] Escobar-Molina R Rodríguez-Ruiz S Gutiérrez-García C Franchini E. Weight loss and psychological-related states in high-level judo athletes. Int J Sport Nutr Exerc Metab. (2015) 25(2):110–8. 10.1123/ijsnem.2013-0163 25029701

[6] Lamas L Heiner M Ferreira M Moura A Rangel W Fellingham G No-gi Brazilian Jiu-Jitsu: a markovian analysis of elite-level combat dynamics. Int J Sports Sci Coach. (2023) 19(4):1767–75. 10.1177/17479541231210979

[7] Jacobson J Matthaeus L. Athletics and executive functioning: how athletic participation and sport type correlate with cognitive performance. Psychol Sport Exerc. (2014) 15(5):521–7. 10.1016/j.psychsport.2014.05.005

[8] Kraemer WJ Fry AC Rubin MR Triplett-McBride T Gordon SE Koziris LP Physiological and performance responses to tournament wrestling. Med Sci Sports Exerc. (2001) 33(8):1367–78. 10.1097/00005768-200108000-00019 11474340

[9] Lucia S Bianco V Boccacci L Di Russo F. Effects of a cognitive-motor training on anticipatory brain functions and sport performance in semi-elite basketball players. Brain Sci. (2021) 12(1):68. 10.3390/brainsci12010068 35053809 PMC8773627

[10] Blick BC. Empathy, mindfulness, and resilience differences across five Brazilian Jiu-Jitsu levels (Doctoral dissertation). Phoenix, Arizona: Grand Canyon University (2024).

[11] Power S. Brazilian Jiu-Jitsu and mental health of law enforcement officers (Doctoral dissertation). Kirkland: Northwest University, College of Social and Behavioral Sciences.

[12] Dos Santos MAF Maurício CDA Soto DAS Aedo-Muñoz E Brito CJ Pierantozzi E Influence of Brazilian Jiu-Jitsu belt graduations on the performance of elite female combat athletes. Pol J Sport Tour. (2024) 31(1):24–30. 10.2478/pjst-2024-0004

[13] Voss MW Kramer AF Basak C Prakash RS Roberts B. Are expert athletes ‘expert’ in the cognitive laboratory? A meta-analytic review of cognition and sport expertise. Appl Cognit Psychol. (2010) 24(6):812–26. 10.1002/acp.1588

[14] Neumann DL Moffitt RL Thomas PR Loveday K Watling DP Lombard CL A systematic review of the application of interactive virtual reality to sport. Virtual Real. (2018) 22:183–98. 10.1007/s10055-017-0320-5

