# Supplementary material for: Cognitive functions and skill level in Brazilian Jiu-Jitsu: an exploratory study using virtual reality
Source: Front Sports Act Living. 2026 Jan 12;7:1738534. doi: 10.3389/fspor.2025.1738534 (PMC12833417; doi:10.3389/fspor.2025.1738534)
Supplement: Supplementary file 1 [file Datasheet1.pdf]

Outcome	Group	Median	Q1	Q3	IQR
1—Reaction time (ms)	Prof	315	305	330	25
1—Reaction time (ms)	Semi	323	319	328	9
1—AVG time to correct decision (ms)	Prof	70	60	75	15
1—AVG time to correct decision (ms)	Semi	230	200	260	60
2—Caught balls (%)	Prof	49	47	52	5
2—Caught balls (%)	Semi	35	25	45	20
2—Release time (ms)	Prof	740	650	820	170
2—Release time (ms)	Semi	710	620	780	160
3—Covered game field (%)	Prof	29,8	26,5	33,1	6,6
3—Covered game field (%)	Semi	28,7	25,4	31,9	6,5
3—Median motor time (ms)	Prof	940	780	1100	320
3—Median motor time (ms)	Semi	1040	940	1120	180
3—Median reaction time	Prof	232	220	245	25
3—Median reaction time	Semi	280	240	320	80
3—Correct attempts (%)	Prof	98	97	99	2
3—Correct attempts (%)	Semi	96	95	97	2
4—Median reaction time	Prof	420	405	435	30
4—Median reaction time	Semi	392	380	425	45
4—Successful tasks (%)	Prof	93	90	96	6
4—Successful tasks (%)	Semi	90	88	94	6
4—Median motor time	Prof	590	520	680	160
4—Median motor time	Semi	168	167	168	1
5—Efficiency (%)	Prof	90	88	95	7
5—Efficiency (%)	Semi	78	72	83	11
5—Concentration (%)	Prof	60	40	95	55
5—Concentration (%)	Semi	15	8	25	17
5—Reaction time (ms)	Prof	315	305	330	25
5—Reaction time (ms)	Semi	323	319	328	9
